# Supplementary material for: The causal relationship between the human gut microbiota and pyogenic arthritis: a Mendelian randomization study
Source: Front Cell Infect Microbiol. 2024 Nov 26;14:1452480. doi: 10.3389/fcimb.2024.1452480 (PMC11629706; doi:10.3389/fcimb.2024.1452480)
Supplement: Supplementary file 1 [file DataSheet1.zip › mendelian test/ebi-a-GCST90027557.csv_forest.pdf]

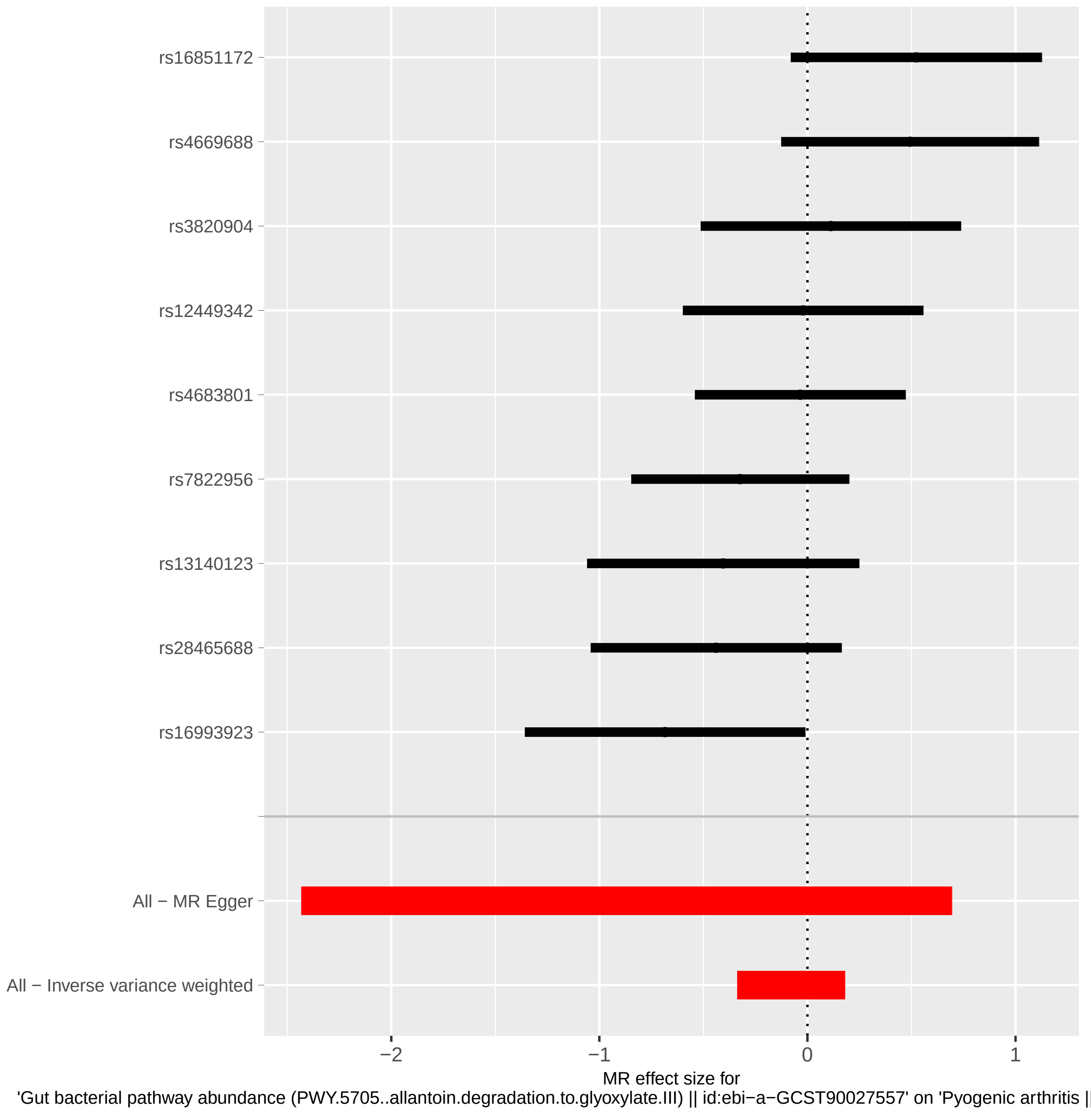
rs16851172
rs4669688
rs3820904
rs12449342
rs4683801
rs7822956
rs13140123
rs28465688
rs16993923
All − MR Egger
All − Inverse variance weighted
−2
−1
0
1
MR effect size for
'Gut bacterial pathway abundance (PWY.5705..allantoin.degradation.to.glyoxylate.III) || id:ebi−a−GCST90027557' on 'Pyogenic arthritis |
